# Lysyl oxidase–like 2 (LOXL2)–mediated cross-linking of tropoelastin

**DOI:** 10.1096/fj.201801860RR

**Published:** 2019-01-24

**Authors:** Christian E. H. Schmelzer, Andrea Heinz, Helen Troilo, Michael P. Lockhart-Cairns, Thomas A. Jowitt, Marion F. Marchand, Laurent Bidault, Marine Bignon, Tobias Hedtke, Alain Barret, James C. McConnell, Michael J. Sherratt, Stéphane Germain, David J. S. Hulmes, Clair Baldock, Laurent Muller

**Affiliations:** * Fraunhofer Institute for Microstructure of Materials and Systems (IMWS), Halle (Saale), Germany;; † Institute of Pharmacy, Martin Luther University Halle-Wittenberg, Halle (Saale), Germany;; ‡ Department of Pharmacy, University of Copenhagen, Copenhagen, Denmark;; § Wellcome Trust Centre for Cell-Matrix Research, University of Manchester, Manchester, United Kingdom;; ¶ Division of Cell-Matrix Biology and Regenerative Medicine, School of Biological Sciences, Faculty of Biology, Medicine and Health, University of Manchester, Manchester, United Kingdom;; ‖ Center for Interdisciplinary Research in Biology (CIRB), Collège de France, CNRS, INSERM, PSL Research University, Paris, France;; # Collège Doctoral, Sorbonne Université, Paris, France;; ** UMR 5305, Centre National de la Recherche Scientifique (CNRS), Laboratoire de Biologie Tissulaire et Ingénierie Thérapeutique (LBTI), Université de Lyon, Lyon, France

**Keywords:** matrix remodeling, elastin, protein structure, SAXS, proteomics

## Abstract

Lysyl oxidases (LOXs) play a central role in extracellular matrix remodeling during development and tumor growth and fibrosis through cross-linking of collagens and elastin. We have limited knowledge of the structure and substrate specificity of these secreted enzymes. LOXs share a conserved C-terminal catalytic domain but differ in their N-terminal region, which is composed of 4 repeats of scavenger receptor cysteine-rich (SRCR) domains in LOX-like (LOXL) 2. We investigated by X-ray scattering and electron microscopy the low-resolution structure of the full-length enzyme and the structure of a shorter form lacking the catalytic domain. Our data demonstrate that LOXL2 has a rod-like structure with a stalk composed of the SRCR domains and the catalytic domain at its tip. We detected direct interaction between LOXL2 and tropoelastin (TE) and also LOXL2-mediated deamination of TE. Using proteomics, we identified several allysines together with cross-linked TE peptides. The elastin-like material generated was resistant to trypsin proteolysis and displayed mechanical properties similar to mature elastin. Finally, we detected the codistribution of LOXL2 and elastin in the vascular wall. Altogether, these data suggest that LOXL2 could participate in elastogenesis *in vivo* and could be used as a means of cross-linking TE *in vitro* for biomimetic and cell-compatible tissue engineering purposes.—Schmelzer, C. E. H., Heinz, A., Troilo, H., Lockhart-Cairns, M.-P., Jowitt, T. A., Marchand, M. F., Bidault, L., Bignon, M., Hedtke, T., Barret, A., McConnell, J. C., Sherratt, M. J., Germain, S., Hulmes, D. J. S., Baldock, C., Muller, L. Lysyl oxidase–like 2 (LOXL2)–mediated cross-linking of tropoelastin.

Extracellular matrix (ECM) remodeling plays a central role in morphogenesis and development as well as in tissue responses to pathologies, including fibrosis and ischemic vascular diseases. The ECM provides cells with mechanical and chemical cues, and its remodeling involves both proteolysis of pre-existing ECM and synthesis of a new microenvironment, as regulated by expression and activity of enzymes responsible for post-translational modifications. Acquiring a better understanding of these modifications and developing new tools to address them is also an issue in the context of tissue engineering because natural polymers are gaining interest as biomaterials for stem cell–based therapeutic approaches. Biomimicking approaches using recombinant enzymes involved in ECM remodeling are thus primary candidates for the production of cell-encapsulating biomaterials.

Cross-linking of ECM proteins is a major step in microenvironment remodeling because this modulates both tensile strength and elastic properties depending on the substrate (*i.e.*, collagens and elastin, respectively). These post-translational modifications are performed by transglutaminases and lysyl oxidases (LOXs), whose specific substrates remain to be determined. LOXs share a conserved C-terminal amine oxidase catalytic domain consisting of a copper-binding motif and a lysine tyrosylquinone cofactor (reviewed in refs. 1–3). They catalyze the oxidative deamination of the ε-amino group of lysines and hydroxylysines in collagens and elastin. The resultant highly reactive aldehydes eventually condense with other oxidized groups or unaltered lysine residues to form a variety of cross-links. The LOX family is composed of 2 subgroups containing LOX and LOX-like (LOXL) 1 and LOXL2–4, respectively. The C-terminal catalytic domains of LOX and LOXL1 share a homology of 88%, and those of LOXL2–4 share a homology of 84–86%, whereas the homology between these 2 groups is only 64–68% [reviewed by Moon *et al*. (2)]. The N-terminal domains of LOX and LOXL1 are shorter than the catalytic domain and act as prodomains, regulating activation and activity of the catalytic domain, whereas the N-terminal region of LOXL2–4 consists of 4 adjacent scavenger receptor cysteine-rich (SRCR) domains, which make up two-thirds of the full-length protein. Their functions remain to be determined. Indeed, processing of the N-terminal domain of LOXL2 between SRCR domains 2 and 3 was recently described as having no impact on enzyme activation, *per se* (4). Although the specific substrates of each member of the family are not yet known, gene inactivation of LOX and LOXL1 has provided interesting clues concerning the natural substrates of these enzymes and the tissues where they are modified (5–8).

LOXL2 was proposed to cross-link collagen IV (9, 10), and we have demonstrated the regulation of angiogenesis by LOXL2 (9). LOXL2 also regulates chondrocyte differentiation, suggesting that it is a multifunctional enzyme (11). There is in addition an extensive literature concerning its involvement in the maintenance of pathologic environments (12), including fibrosis, tumor progression, and metastasis dissemination, that suggested that LOXL2 cross-links fibrillar collagens. LOXL2 is indeed considered an important therapeutic target in heart failure and fibrosis (13). We, however, still lack investigations of LOXL2-mediated oxidation of other collagens and elastin [reviewed by Moon *et al*. (2)]. The first patients carrying LOXL2 mutations were recently identified as suffering from middermal elastolysis (14), raising the possibility that LOXL2 could participate in elastin cross-linking. In the present study, we first determined the nanoscale structure of both full-length LOXL2 (FL-LOXL2) and a catalytic domain–deleted form that only comprises the 4 SRCR domains (SRCR14). We then investigated interactions with tropoelastin (TE) and the ability of LOXL2 to oxidize and cross-link TE. We measured the level of LOXL2-generated desmosine and found that cross-linking of coacervated TE affects both its stiffness and resistance to proteolytic degradation, thus suggesting the involvement of LOXL2 in elastin biosynthesis and making it a promising tool for the fabrication of elastin-based biomaterials.

## MATERIALS AND METHODS

### Protein purification

cDNA encoding human FL-LOXL2 or a truncated form consisting of SRCR14 was inserted into pcDNA3.1-*myc*-His at the HindIII and XbaI restriction sites. PCR fragments were generated using the following primers: 5′-AAGCTTATGGAGAGGCCTCTG-3′ and 5′-TCTAGACTGCGGGGACAGCTG-3′ for FL-LOXL2 and 5′-TCTAGAGGTTTCTGAGCAGGC-3′ for SRCR14. Dihydrofolate reductase (DHFR)–deficient Chinese hamster ovary (CHO) cells were cotransfected with the *dhfr* gene and constructs encoding FL-LOXL2 or SRCR14 using Lipofectamine 2000 according to the manufacturer’s recommendations (Thermo Fisher Scientific, Waltham, MA, USA). High-expression clones were selected by serial subcloning in the presence of increasing concentrations of methotrexate (MilliporeSigma, Burlington, MA, USA). Transfected CHO cells were cultured in Opti-MEM (Thermo Fisher Scientific) for protein production. Purification was performed on HisTrap columns (GE Healthcare, Waukesha, WI, USA). Protein was eluted at 200 mM imidazole, and buffer was exchanged for 50 mM Na phosphate buffer (pH 7.4) containing 150 mM NaCl by gel filtration.

Recombinant human FLAG-tagged bone morphogenetic protein-1 (BMP-1) (in 20 mM Na-HEPES, 0.5 M NaCl, 2.5 mM CaCl_2_, and 0.1% *n*-octylglucoside, pH 7.4) and His-tagged procollagen C-proteinase enhancer 1 (PCPE-1) (in 20 mM Na-HEPES, 0.5 M NaCl, and 5 mM CaCl_2_, pH 7.4) were produced in 293- Epstein–Barr nuclear antigen (EBNA) cells as described in Blanc *et al*. (15). The control substrate CPIII-Long (in 20 mM HEPES, 0.5 M NaCl, and 5 mM CaCl_2_, pH 7.4) was produced in 293-EBNA cells as described in Bourhis *et al.* (16).

Human TE, isoform 2 was produced in an *Escherichia coli* expression system based on refs. 17 and 18 with some modifications. In brief, bio wet mass was heated in a microwave to inactivate intrinsic proteases. Cell disintegration was carried out using a high-pressure homogenizer. After centrifugation, TE was extracted twice from the pellet using an acetone and buffer mixture. The combined extracts were evaporated and concentrated by partial removal of water, and the turbid suspension was filtered. The precipitated TE on the filter membrane was solubilized by the addition of buffer and decolorized by the addition of charcoal. After filtering off the charcoal, the TE solution was dialyzed twice against distilled water. In the last step, pure TE was isolated by freeze drying.

Bovine aortic elastin and human aortic elastin were isolated from aortic tissue as previously described in Schmelzer *et al.* (19). Analytical-grade Tris and formic acid were purchased from Merck (Darmstadt, Germany). HPLC-grade acetonitrile was obtained from VWR International (West Chester, PA, USA), and TFA was obtained from MilliporeSigma.

### Proteolysis experiments

Proteolytic processing of LOXL2 produced by CHO cells was analyzed in overnight secretion medium prepared in the presence of different serine protease inhibitors [aprotinin, leupeptin, and 4-(2-aminoethyl)benzenesulfonyl fluoride hydrochloride]. Proteins were solubilized with Laemmli buffer, and LOXL2 was detected by Western blotting using anti-LOXL2 from Abnova (Taipei City, Taiwan). To test for possible cleavage of LOXL2 by BMP-1, 100 µl of purified FL-LOXL2 was first dialyzed extensively against assay buffer (50 mM Na-HEPES, pH 7.4; 150 mM NaCl; 5 mM CaCl_2_) at 4°C overnight. Reactions were set up containing 2.8 µg/ml (20 nM) BMP-1, 25 µg/ml (150 nM) FL-LOXL2, or 48 µg/ml (200 nM) CPIII-Long [used as a positive control (16)] with or without 200 nM PCPE-1, with Brij 35 (Thermo Fisher Scientific) added to a final concentration of 0.02% (w/v) in low-binding Eppendorf tubes (Eppendorf, Hamburg, Germany) for 2 h at 37°C. Reactions were stopped by transfer to ice and addition of EDTA and 5× Laemmli sample to a final concentration of 50 mM EDTA, followed by heating to 100°C for 3 min. Samples were analyzed by SDS-PAGE using 4–20% gradient gels (Bio-Rad, Hercules, CA, USA) and staining with Coomassie Blue (MilliporeSigma).

Human TE, cross-linked TE (cTE), and aortic elastin were weighed and dissolved or dispersed at a concentration of 5 mg/ml in 50 mM Tris-HCl, pH 7.5. Digestions with cathepsin G (CG; Elastin Products Company, Owensville, MO, USA), trypsin (Serva Electrophoresis, Heidelberg, Germany), and porcine pancreatic elastase (PE; Elastin Products Company) were carried out at 37°C for 24 h at enzyme:substrate ratios of 1:100 (w/w), 1:100 (w/w), and 1:50 (w/w), respectively. All digestions were stopped by addition of TFA to a final concentration of 0.5% (v/v), and then samples were stored at −26°C until mass spectrometry (MS) analysis.

### Multiangle light scattering, analytical ultracentrifugation, and circular dichroism

Samples (0.5 ml at ∼0.5 mg/ml) in 50 mM Na phosphate and 500 mM NaCl, pH 7.4 were injected onto a gel filtration column (Superdex 200 for FL-LOXL2 or Superdex 75 for SRCR14; GE Healthcare) at 0.75 ml/min. For multiangle light scattering, samples were passed through a Dawn Heleos II 18-angle laser photometer (Wyatt Technology, Santa Barbara, CA, USA). This was coupled to an Optilab rEX refractive index detector (Wyatt Technology), and data were analyzed using Astra 6 software (Wyatt Technology). Sedimentation velocity analytical ultracentrifugation (AUC) was carried out at 45,000 rpm at 20°C using an XL-A ultracentrifuge with an An-50 Ti 4-Hole Rotor (Beckman Coulter, Brea, CA, USA). Sedimentation was scanned every 90 s for 200 scans. Data were analyzed using Sedfit (*https://sedfitsedphat.nibib.nih.gov/software/default.aspx*).

Prior to analysis by circular dichroism (CD), samples of recombinant LOXL2 and SRCR14 were further purified by gel filtration using a Superdex 200 10/300 column pre-equilibrated in 50 mM Na phosphate and 150 mM NaCl, pH 7.4. Peak fractions were analyzed by SDS-PAGE using 4–20% gradient gels in nonreducing conditions. Far UV (192–260 nm) CD measurements were carried out using thermostated 0.2-mm path length quartz cells in an Applied Photophysics (Leatherhead, United Kingdom) Chirascan instrument [Protein Science Facility, Structure Fédérative de Recherche (SFR) Biosciences Unité Mixte de Service (UMS) 3444/Unité de Service (US) 8, Lyon, France]. Proteins (∼200 µg/ml) were analyzed at 25°C in the same buffer. Spectra were measured in duplicate using a wavelength increment of 1 nm, an integration time of 8 s, and a bandpass of 1 nm. Protein concentrations were determined by Nanodrop (Thermo Fisher Scientific). Observed ellipticities were converted to mean residue molar ellipticities, taking into account mean residue weight, cell path length, and protein concentration.

### Small-angle X-ray scattering

For small-angle X-ray scattering (SAXS), samples of FL-LOXL2 and SRCR14 in the concentration range 3–6 mg/ml were prepared in 50 mM Na phosphate and 0.15 M NaCl, pH 7.4. Before loading, samples were centrifuged for 10 min at 12,000 *g*, and the supernatants were collected. Initial data collection was carried out on beamline B21 at Diamond Light Source (Didcot, United Kingdom) using the in-house BioSAXS robot. This revealed the presence of small amounts of aggregated material in both specimens at these high concentrations. To completely eliminate aggregates, further experiments were carried out at the European Synchrotron Radiation Facility (ESRF; Grenoble, France) on beamline B29 using in-line size-exclusion chromatography (Superdex 200 Increase 3.2/300). Prior to injection, samples were concentrated to ∼6 mg/ml using Vivaspin 500 µl 10-kDa concentrators (Vivaproducts, Littleton, MA, USA). SAXS data were collected throughout elution from the column at 1-s intervals, and the radius of gyration (*R*_g_) was automatically calculated for each scan. Scans throughout the elution peak with constant *R*_g_ values were then pooled and used for subsequent structure modeling. Data were processed using the PRIMUS (20) and GNOM (21) programs. Low-resolution structures were then modeled using the programs DAMMIF (22) and MONSA (23). Rigid-body modeling was performed to the SAXS data with CORAL (23) using homology models of the first and second SRCR domains and the structures of SRCR domains 3, 4, and the catalytic domain (24).

### Electron microscopy single-particle reconstruction

FL-LOXL2 (10 µg/ml) was first absorbed onto glow-discharged carbon-coated grids and stained with 4% uranyl acetate (pH 4.7). Images were recorded at ×23,000 on a Tecnai Biotwin (Field Electron and Ion Company; Thermo Fisher Scientific) at 120 kV with an Orius CCD camera (Gatan, Pleasanton, CA) with a 1-s exposure at 0.5–1.5 µm defocus range at 2.8 Å/pixel. Using EMAN2 (25), 10,000 particles were picked using a combination of manual and semiautomated picking. The contrast transfer function was corrected, and the dataset was subjected to 2-dimensional classification. These classes were used to generate an initial 3-dimensional (3D) model to seed 6 rounds of iterative refinement to produce a self-consistent 3D structure. Data were split into odd and even numbers, and each dataset was refined independently to produce a resolution curve for each stage of refinement. With a Fourier shell correlation cutoff value of 0.143, the resolution estimate was 22 Å. Modeling was performed using University of California–San Francisco (UCSF) Chimera (26).

### Surface plasmon resonance

Protein-protein interactions were measured with a ProteOn XPR36 (Bio-Rad) in 10 mM Na-HEPES, 150 mM NaCl, 2 mM CaCl_2_, and 0.05% Tween 20, pH 7.4 (running buffer) at 25°C. Proteins were immobilized on a GLC chip (Bio-Rad) *via* standard amine coupling with 1-Ethyl-3-(3-dimethylaminopropyl)carbodiimide (EDS)/N-hydroxysulfosuccinimide (s-NHS) using 10 µg/ml LOXL2 diluted in 100 mM acetate buffer, pH 5.0 at 100 µl/min for 60 s. Free reactive N-hydroxysulfosuccinimide (NHS) esters were blocked using ethanolamine. A reference lane was activated and blocked with ethanolamine but with no LOXL2 added. The chip was rotated by 90°, and analytes were flowed over the LOXL2 and reference sensors in sequential lanes using a serial 1:1 dilution from 830 nM TE at a flow rate of 100 µl for 120 s with a dissociation time of 300 s. Regeneration of the LOXL2 was achieved using a 30-s pulse of 50 mM NaOH.

### LOX activity and cross-linking experiments

LOXL2 activity was measured using the Amplex UltraRed assay (Thermo Fisher Scientific) as previously described in Trackman and Bais (3). For this, 4 µg LOXL2 was assayed in 50 mM Na borate, pH 8.2 in the presence of 10 mM cadaverin (MilliporeSigma), 10 µM Amplex UltraRed, and 1 U/ml horseradish peroxidase (MilliporeSigma). LOXL2 activity was also measured using 1.25 µM laminin or 1.65 µM TE. Resorufin fluorescence was measured at 37°C for 60 min using an EnVision Xcite multimode plate reader (PerkinElmer, Waltham, MA, USA) at λ_ex_ = 535 nm and λ_em_ = 610 nm. The amount of H_2_O_2_ released by LOXL2 was determined using a standard curve of hydrogen peroxide. β-Aminopropionitrile (β-APN) (500 µM) was added to the reaction mixture in some experiments. Measurements were performed in triplicate, and a representative experiment is shown.

TE was dissolved in 50 mM Na borate buffer, pH 8.0 at a concentration of 10 mg/ml, then incubated at 50°C for 15 min to induce coacervation. LOXL2 was then added at an enzyme:substrate ratio of 1:500 (w/w), and the mixture of enzyme and substrate was incubated at 50°C for 24 h. The precipitated insoluble cTE that had formed was washed with a solution of water and ethanol (1:1, v/v), dried in a SpeedVac (Thermo Fisher Scientific), and stored at −26°C prior to further analysis.

### Mass spectrometric analysis and peptide identification

Trypsin digests were analyzed using an Ultimate 3000 RSLCnano System (Thermo Fisher Scientific) coupled to a Q-Tof II Mass Spectrometer (Waters, Milford, MA, USA) as previously described in Heinz *et al.* (27). CG and PE digests were separated on an Ultimate 3000 RSLCnano System coupled to an Orbitrap Fusion Tribrid mass spectrometer (Thermo Fisher Scientific) equipped with a Nanospray Flex Ion Source (Thermo Fisher Scientific). Peptide mixtures were loaded on the trap column (Acclaim PepMap RP-C18, 300 μm × 5 mm, 5 μm, 100 Å; Thermo Fisher Scientific) and washed with water containing 0.1% TFA for 15 min at the rate of 30 μL/min before the peptides were separated on the separation column (Acclaim PepMap RP-C18, 75 μm × 250 mm, 2 μm, 100 Å) at a flow rate of 300 nl/min using gradients from 1 to 35% solvent B for 90 min and 35–85% B for 5 min followed by 85% B for 5 min. Solvent B was acetonitrile containing 0.1% formic acid, and solvent A was water containing 0.1% formic acid. Data were acquired using the data-dependent tandem MS (MS/MS) mode. Each high-resolution full scan in the Orbitrap (*m/z* 300–1500, *R* = 120,000) was followed by high-resolution product ion scans in the Orbitrap (higher-energy collisional dissociation mode with 27% normalized collision energy, *R* = 15,000) within 5 s, starting with the most intense signal in the full-scan mass spectrum. Dynamic exclusion was enabled to allow analysis of less-abundant ions. Data acquisition was controlled with Xcalibur 3.0.63 (Thermo Fisher Scientific). The raw data of the PE and CG digests were refined and analyzed using Peaks 7.5 (Bioinformatics Solutions, Waterloo, ON, Canada), and the human subsection of the Swiss-Prot database was used for database matching. The mass error tolerance for precursor and fragment ions was set to 2.0 ppm and 0.015 Da, respectively. The enzyme was set to “none,” and oxidation of K residues to peptidyl α-aminoadipic-σ-semialdehyde (allysine) and deamidation of Q and N were taken into account as variable modifications. Intramolecular cross-links were detected either by using the program StavroX *(https://www.stavrox.com*) (28) or with Peaks by applying a database with a modified sequence of human TE. Briefly, cross-linking motifs were reduced to a single K residue in the sequence, and the mass differences between this residue and the corresponding intramolecularly cross-linked motif were included as variable post-translational modifications in a separate search.

### Detection and quantification of cross-links

All elastin samples were totally hydrolyzed and further treated and analyzed as previously described in Heinz *et al.* (27). In brief, liquid chromatography (LC)–MS analysis of desmosine and isodesmosine was carried out using an Agilent 1100 LC system (Agilent Technologies, Santa Clara, CA, USA) coupled to a quadrupole ion trap mass spectrometer (Finnigan LCQ; Thermo Fisher Scientific) with an electrospray interface operated in the positive ion mode.

### Atomic force microscopy

Samples were rehydrated in distilled water and left to adhere to a charged glass slide overnight at room temperature. They were then rehydrated in distilled water for micromechanical assessment by atomic force microscopy (AFM) indentation using a Bioscope Catalyst AFM (Bruker, Billerica, MA, USA) mounted onto an Eclipse T1 inverted optical microscope (Nikon, Tokyo, Japan) fitted with a spherically tipped cantilever (nominal radius and spring constant of 1 μm and 3 N/m, respectively; Windsor Scientific, Slough, United Kingdom) running Nanoscope Software 8.15 (Bruker). The local reduced modulus was determined for each of 25 points in a 50-µm × 50-µm region, indented at a frequency of 1 Hz with lateral spacing of 2 µm. The approach force curve was used to calculate the reduced modulus using the Hertzian contact mechanics model and a 70% force fit.

### Physicochemical characterization of cTE

Differential scanning calorimetry was carried out to determine the glass transition temperature (*T*_g_) of cTE and bovine aortic elastin. Experiments were performed using a differential scanning calorimeter (DSC 200; Netzsch-Gerätebau, Selb, Germany). For this, 3 mg of dried samples was placed in aluminum pans and covered by pierced lids. Samples were heated from 30 to 250°C at 20°C/min followed by cooling from 250 to 30°C at the same rate. This program was repeated twice for each sample. The onset of the *T*_g_ was determined using the DSC 200 software. Swelling properties of cTE and bovine aortic elastin were determined at 4 and 37°C by immersing samples in water. The samples were taken out after 2 h and weighed after removal of surface-bound water. All measurements were carried out in triplicate. The swelling ratio in milligrams of water per milligram of protein (*i.e.*, the water absorptivity of samples) was calculated using the following equation:


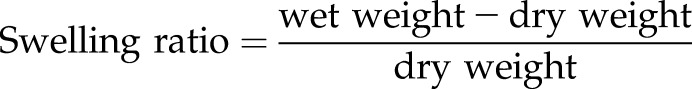


### Analysis of LOXL2 expression

For immunohistochemistry, aortas from 6-wk-old C57BL/6 mice were embedded in cryoblock medium (VWR International) on dry ice. Cryosections were incubated with anti-LOX (Abcam, Cambridge, MA, USA) or anti-LOXL2 (Santa Cruz Biotechnology, Dallas, TX, USA) before fixation with acetone and further processing with secondary antibodies coupled to Alexa Fluor fluorochromes (Thermo Fisher Scientific) as previously described in Bignon *et al.* (9).

Frozen aortas were pulverized on dry ice before lysis in 25 mM Tris (pH 7.5), 100 mM NaCl, 5 mM EDTA, and 1% Triton X-100 containing Protease Inhibitor Cocktail (MilliporeSigma). Samples were centrifuged for 30 min at 15,000 *g,* and pellets were solubilized in Laemmli buffer. Proteins of both lysates were separated by SDS-PAGE and electrotransfered to PVDF membranes for immunodetection of LOX, LOXL2 (Abnova), fibronectin (Merck), and actin (Abcam).

## RESULTS

### Production and preliminary biophysical characterization of recombinant human LOXL2

**Figure 1:**
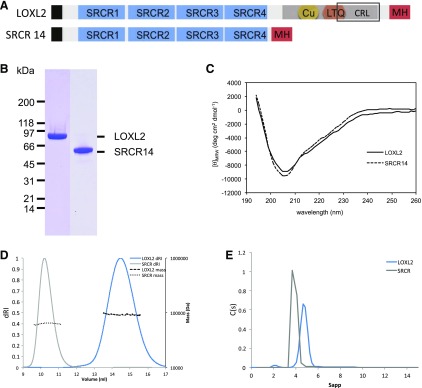
Characterization of recombinant human FL-LOXL2 and the SRCR14 region. *A*) Schematic representation of recombinant FL-LOXL2 and of the catalytic domain truncated form (SRCR14). Black box corresponds to the signal peptide. Dark gray box corresponds to the catalytic domain, containing the copper binding site (Cu), the lysine tyrosylquinone cofactor (LTQ), and the cytokine receptor–like domain (CRL, in black box). The C-terminal myc-histidine tag (MH) is in red. *B*, *C*) Purified recombinant proteins were separated by SDS-PAGE and stained with Coomassie Blue (*B*) and analyzed by CD (*C*). *D*, *E*) Purity and MWs were also assessed using multiangle laser light scattering AUC. Multiangle laser light scattering was performed with a Superdex 200 column for LOXL2 and a Superdex 75 for SRCR14 (*D*). Ultracentrifugation was analyzed using Sedfit2 (*E*). dRI, differential refractive index; C(s), sedimentation coefficient distribution; Sapp, apparent sedimentation; deg, degrees; MRW = mean residue weight.

For large-scale production of recombinant human LOXL2, we selected eukaryotic CHO cell lines expressing high levels of His-tagged LOXL2 according to their resistance to inhibition of DHFR by methotrexate. When cultured for more than 4 d at high density in roller bottles, these cells released LOXL2 as 2 bands on SDS-PAGE of ∼100 and ∼65 kDa with similar intensities, unless serine protease inhibitors were included in the medium (Supplemental Fig. S1*A*). Detection of these 2 bands after Ni–nitrilotriacetic acid affinity enrichment for the C-terminally His-tagged LOXL2 suggested that SRCR domains 1 and 2 were released by cleavage of LOXL2 between SRCR domains 2 and 3, as recently proposed in López-Jiménez *et al.* (4). Because processing of LOX and LOXL1 by BMP-1 is involved in their proteolytic activation (29–31), we investigated *in vitro* whether LOXL2 was also a substrate of this protease. No processing of LOXL2 by BMP-1 was observed, even in the presence of the enhancer PCPE-1 (Supplemental Fig. S1*B*). CHO cells transfected with a plasmid encoding either FL-LOXL2 or a shorter form lacking the catalytic domain (called SRCR14 because it encompasses the N-terminal SRCR domains 1 to 4) (**Fig. 1*A***) were thus cultured in serum-free medium containing 5 µM aprotinin. Recombinant proteins were affinity purified and analyzed by Coomassie Blue staining and CD (Fig. 1*A–C*). Both proteins gave typical β-sheet spectra consistent with the known structure of the SRCR domain (32). Multiangle light scattering and AUC (Fig. 1*D, E* and **Table 1**) revealed that FL-LOXL2 and SRCR14 had MWs of 92.6 ± 2.8 kDa (93 kDa predicted) and 63.6 ± 2 kDa (65 kDa predicted), respectively, with hydrodynamic radii corresponding to very extended structures, as also indicated by dynamic light scattering (unpublished results).

**TABLE 1:** Structural characteristics of LOXL2 and SRCR14: comparison of the *R*_g_, hydrodynamic radius, frictional ratio, and maximum dimension calculated from MALS, AUC, SAXS, and TEM data

Variable	MALS		AUC		SAXS		TEM
	LOXL2	SRCR14	LOXL2	SRCR14	LOXL2	SRCR14	LOXL2
MW (kDa)	92.6	63.6					93
*R*_h_ (nm)	5.6	4.35	5.8	4.1			6.6
*R*_g_ (nm)					4.35	3.64	4.7
S_20,W_			5.15	4.18			5.52
f/f0			1.44	1.38			
D_max_ (nm)					17.1	12.9	17.6

D_max_, maximum dimension; f/f0, frictional ratio; MALS, multiangle light scattering; *R*_h_, hydrodynamic radius; S_20,W_, sedimentation coefficient; *R*_g_, radius of gyration; TEM, transmission EM.

### Nanoscale structure of recombinant human LOXL2

**Figure 2:**
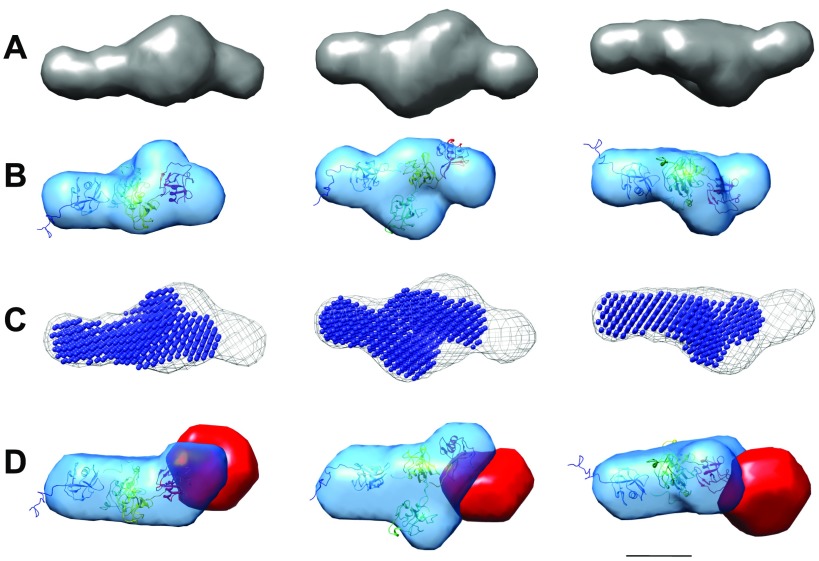
Low-resolution structures and modeling of LOXL2 and SRCR by SAXS analysis. *A*) DAMMIF *ab initio* models of LOXL2. *B*) The SRCR DAMMIF *ab initio* model is superimposed with the rigid-body model of SRCR14 colored from blue to red. *C*) The *ab initio* model of the SRCR region (blue) inside the *ab initio* model of LOXL2 (mesh). *D*) MONSA model of LOXL2 with the catalytic domain shown as a solid red density and the SRCR domains shown in blue, into which the rigid-body model of the SRCR domains is docked (shown colored from blue to red). Scale bar, 5 nm.

In order to determine the shape and low-resolution structure of LOXL2, we performed SAXS experiments using in-line gel filtration at the ESRF. Both SRCR14 and FL-LOXL2 behaved as monodisperse particles in solution, as shown by the linear Guinier plots (Supplemental Fig. S2). Distance distribution plots, calculated using GNOM, confirmed the rod-like shapes of both molecules, with the SRCR domains alone extending over ∼130 Å and the catalytic domain adding an extra ∼40 Å to the length (Supplemental Fig. S2 and Table 1). DAMMIF modeling using the SAXS data allowed determination of the *ab initio* shapes of each protein (**Fig. 2*A, B***). Rigid-body modeling of SRCR14 performed using CORAL was superimposed with the *ab initio* model and showed similar structures (Fig. 2*B*). Superimposition of the SRCR14 *ab initio* model within that of LOXL2 suggested that the catalytic domain projects out at one end of the molecule (Fig. 2*C*). This was confirmed using MONSA, which allowed separate modeling of the catalytic domain using scattering data for FL-LOXL2 and its SRCR14 fragment (Fig. 2*D*).

**Figure 3:**
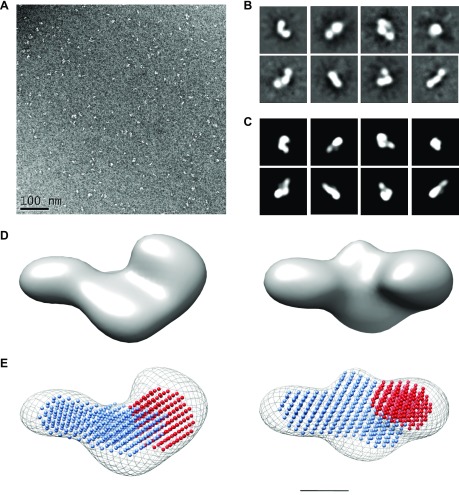
Structural analysis of LOXL2 by single-particle EM and image reconstruction. *A*) Raw image of LOXL2 collected at ×23,000, 2.8 Å/pixel. *B*) Selected class averages of LOXL2. A total of 32 classes were generated from ∼10,000 particles selected from 150 micrographs. *C*) Corresponding projections from the 3D reconstruction generated using EMAN2. Box size is 36 nm (*B*, *C*). *D*) LOXL2 3D reconstruction shown in 2 orientations. *E*) MONSA-SAXS model (catalytic domain shown in red and SRCR region in blue) fitted within the EM model (mesh). Two orientations are shown. Scale bar, 5 nm (*D*, *E*).

The structure of FL-LOXL2 was also investigated by single-particle analysis using transmission electron microscopy (EM) after negative staining (**Fig. 3*A–C***). Approximately 10,000 LOXL2 molecules were selected, and reference-free class averages were generated for 3D reconstruction to 22-Å resolution (Fig. 3*D* and Table 1). Both the monomeric nature of LOXL2 and the overall shape of the protein were confirmed. Indeed, the MONSA-SAXS model could be fitted within the 3D reconstruction of EM (Fig. 3*E*). Altogether, these data suggest that the SRCR catalytic domains are organized in a string of pearls rather than as a globular structure. Again, the structure determined indicated that the catalytic domain is exposed at the tip of a stalk-like structure generated by SRCR14.

**Figure 4:**
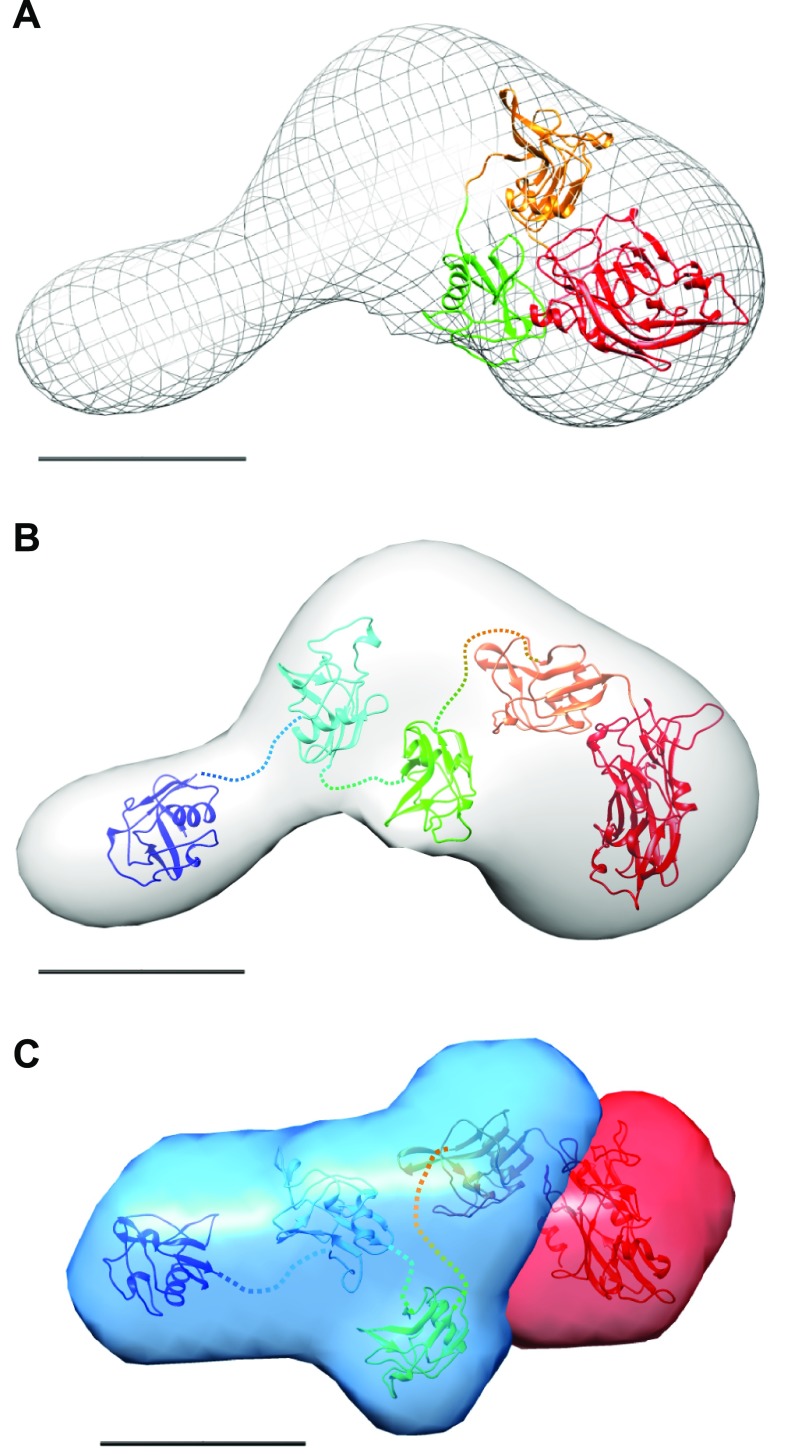
Comparison of SAXS and EM data for FL-LOXL2 with the crystal structure of LOXL2 N455Q lacking SRCR domains 1 and 2. *A*) Crystal structure directly superimposed on the EM model (mesh). *B*, *C*) The 2 rigid-body models that best fit the FL-LOXL2 EM reconstruction (*B*) and MONSA-SAXS model (*C*). SRCR domains are numbered and colored from blue at the N terminus to red at the C terminus. The catalytic domain and SRCR domain 4 from the crystal structure were treated as a rigid body, whereas SRCR domains 1–3 had flexible linkers (represented as dashed lines). The catalytic domain is shown in red and SRCR region in blue (*C*). Scale bars, 5 nm.

A crystal structure of a glycosylation-deficient mutant of LOXL2 lacking the 2 N-terminal SRCR domains was recently described in Zhang *et al.* (24). Comparison with our SAXS and EM data suggested a difference in conformation between full-length and truncated LOXL2 (**Fig. 4*A***). Whereas we found using FL-LOXL2 that the SRCR domains project linearly away from the catalytic domain, the crystal structure of Zhang *et al.* (24) indicates that the SRCR domain 3 folds back on the catalytic domain. The presence of large linkers (9–33 residues) between the SRCR domains adds flexibility to the organization of these domains. Indeed, the best-fitting models to the SAXS and EM data were obtained when SRCR domains 1–3 were considered freely linked domains, with only SRCR4 connected rigidly to the catalytic domain (Fig. 4*B, C*).

### LOXL2-mediated cross-linking of TE

**Figure 5:**
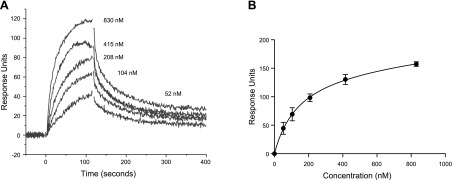
Direct interaction between LOXL2 and TE. *A*) Binding between immobilized LOXL2 was analyzed with TE as analyte using surface plasmon resonance. *B*) Binding constants were determined using steady-state binding analysis, which gives a dissociation constant of 146 ± 16 nM (*n* = 4).

**Figure 6:**
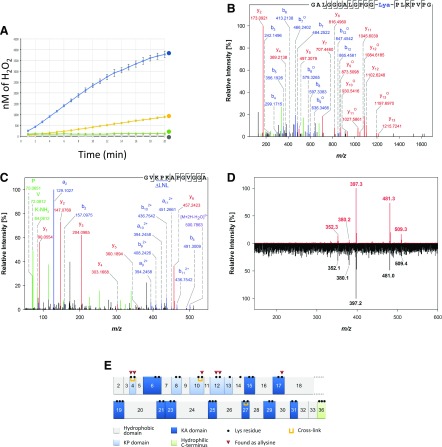
LOXL2-mediated modification of lysines results in cross-linking of TE. *A*) Recombinant LOXL2 activity was measured using the Amplex UltraRed assay using either cadaverine (blue) or TE (yellow) as a substrate. No release of H_2_O_2_ was detected in the presence of 500 µM β-APN (gray) or when using laminin (green) as a substrate. *B*, *C*) Annotated product ion spectra of an allysine (Lya)-containing peptide with *m/z* 849.9803 (z = 2) (*B*) as well as an intramolecularly cross-linked peptide (*m/z* 509.7868; z = 2) possessing a dehydrolysinonorleucine (ΔLNL) (*C*). Both peptides were identified in a digest of cTE. The amino acid sequences are displayed in the upper-right corners of the spectra. Corresponding b-ions and a-ions are highlighted in blue, y-ions are shown in red, and internal fragments are shown in green; product ions denoted with ° indicate loss of water. *D*) Tandem mass spectrum of free desmosine (M^+^ 526.29) detected in a TE sample cross-linked by LOXL2 after total hydrolysis (bottom). The fragment pattern shows clear conformity with a desmosine standard (top) and verifies the presence of desmosine in the sample. *E*) Domain structure of human TE (isoform 2) indicating the identified allysines and cross-linking sites within the sequence. Lys residues are shown as filled circles within Lys-Ala (KA) and Lys-Pro (KP) domains highlighted in dark and light blue, respectively. The domain numbering shown is based on exon assignment, and all domains are displayed in appropriate size corresponding to their relative length. In total, we identified 3 intramolecular bifunctional cross-links and 6 allysine residues.

We then investigated the interactions between LOXL2 and the potential substrate TE. Surface plasmon resonance experiments were performed using immobilized recombinant FL-LOXL2 with TE as the analyte in the mobile phase (**Fig. 5**). We determined a dissociation constant of 146 nM for FL-LOXL2. We thus investigated TE oxidation by FL-LOXL2. LOXL2 activity (7.7 pM/min/µg) was first measured using cadaverin as a substrate, and the Amplex UltraRed assay was used for LOX activity measurements (33). Activity was completely inhibited by β-APN. We then detected oxidation of TE by LOXL2 but not of laminin (**Fig. 6*A***). MS indeed confirmed the presence of allysines in LOXL2-treated TE (Fig. 6*B*), whereas neither allysine nor cross-link were detected in recombinant TE alone. The oxidation of lysine residues resulted in cross-linking of TE, as suggested by the formation of an insoluble, gel-like, elastic material formed at the bottom of the reaction tube during a 24-h incubation (Supplemental Video S1). Optimal cross-linking conditions were determined by varying the temperature and enzyme:substrate ratio, and it was found that 50°C and an enzyme:substrate ratio of 1:500 resulted in the highest yields of 65 ± 6% (*n* = 4) cTE based on the amount of TE used for *in vitro* cross-linking. cTE was further analyzed for the formation of covalent cross-links by MS. The presence of intramolecularly cross-linked peptides was identified by detection of dehydrolysinonorleucine (Fig. 6*C*). In addition, the tetrafunctional cross-link desmosine was also detected after total hydrolysis of cTE in 6 M HCl and subsequent LC–MS/MS analysis on an ion trap instrument (Fig. 6*D*). In agreement with these data, comparison of the results of amino acid analysis of TE and cTE revealed that up to 14% of all lysine residues were either modified to allysine residues or involved in cross-linking in cTE (Supplemental Fig. S3). Hence, compared with aortic elastin, in which about 90% of the lysine residues are cross-linked, lysine residues in cTE were about 6 times less modified. Overall, investigation of peptides in the proteolytic digests of cTE by MS/MS allowed the identification and assignment of 6 free allysine residues and 3 intrapeptidal bifunctional dehydrolysinonorleucine cross-links (Fig. 6*E*).

### Characterization of cTE

**Figure 7:**
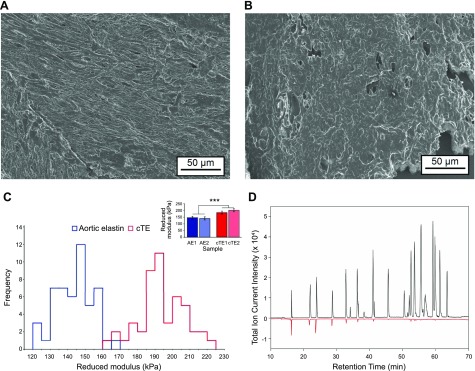
LOXL2-mediated cross-linking of TE results in formation of an insoluble elastin-like material. *A*, *B*) Scanning electron micrographs of isolated aortic elastin (AE) (*A*) and cTE (*B*). *C*) Local micromechanical stiffness was measured by AFM indentation of aortic elastin and cTE. The distribution of reduced modulus values (main panel) was significantly shifted to the right for cTE (red) when compared with aortic elastin (blue). Analysis of mean reduced modulus values from 2 replicates (inset panel) showed that cTE (red; 193.1 ± 13.9 kPa) exhibited a significantly increased reduced modulus when compared with aortic elastin (blue; 144.2 ± 10.8 kPa). ***P < 0.0005. *D*) Total ion current intensity chromatograms obtained by nano–HPLC-MS runs of the 24-h tryptic digests of cTE (red) *vs.* unmodified TE in solution (black).

Scanning EM of cTE revealed a similar appearance when compared with human aortic elastin (**Fig. 7*A, B***). We then investigated the biophysical properties of cTE. The *T*_g_ of cTE was determined to be 160°C, whereas the *T*_g_ of bovine aortic elastin was found to be 180°C (Supplemental Fig. S4*A*). The swelling properties of bovine aortic elastin and cTE were also determined and compared: 1 mg of bovine elastin absorbed 1.7 ± 0.3 mg and 1.3 ± 0.1 mg of water at 4 and 37°C, respectively. In contrast, 1 mg of cTE took up 35.1 ± 2.8 mg and 39.7 ± 8.4 mg of water at 4 and 37°C, respectively (Supplemental Fig. S4*B*). We then characterized the impact of LOXL2-mediated cross-linking of TE on stiffness and resistance to proteolytic digestion. The reduced modulus, a measure of stiffness, was determined by AFM for rehydrated cTE and compared with that of human aortic elastin (Fig. 7*C*). Whereas the human mature elastin had a reduced modulus of 144.2 ± 10.8 kPa, a reduced modulus of 193.1 ± 13.9 kPa was determined for cTE. With regard to proteolytic susceptibility, both bovine aortic elastin and cTE samples were readily degraded by porcine PE and human CG without any remaining insoluble residue, whereas differences were observed in the susceptibility to cleavage by trypsin. Whereas mature elastin was completely resistant to cleavage by trypsin, cTE was found to be partly cleaved. In total, 47% of the insoluble cTE pellet was still present after digestion with trypsin for 24 h, indicating that LOXL2-mediated cross-linking protected TE from proteolytic digestion. In contrast, with non–cross-linked substrate, no TE could be detected 10 min after addition of trypsin. The differences in the proteolytic digests are shown as peptide profiles acquired using HPLC-MS in Fig. 7*D*. The mass spectra of the released peptides from cTE revealed that most were smaller than 2 kDa, whereas larger breakdown products were observed in the TE digest.

### Vascular expression of LOXL2 and elastin

**Figure 8:**
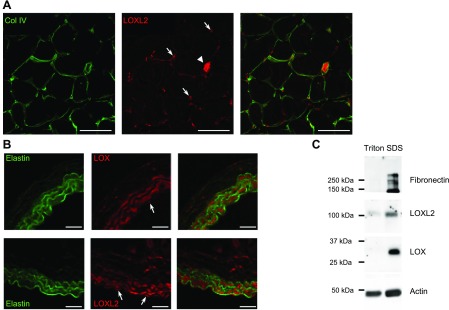
LOXL2 is expressed in the vascular wall. *A*) LOXL2 was detected in cryostat sections of mouse tibialis anterior. Whereas type IV collagen (Col IV) was detected in the basement membrane of both blood vessels and muscle fibers, LOXL2 was restricted to blood vessels (arrows), with a very high expression in arterioles (arrowheads). Scale bars, 50 µm. *B*) LOX and LOXL2 were detected in the intima (arrows) and media of mouse aorta cryosection by immunofluorescence, together with elastin autofluorescence (green). Scale bars, 50 µm. *C*) Aorta were frozen and extracted with Triton X-100 followed by SDS extraction of the insoluble fraction before separation by SDS-PAGE. LOX and LOXL2 were both detected in the Triton X-100–resistant ECM fraction together with fibronectin.

To address the *in vivo* relevance of TE cross-linking by LOXL2, we investigated its distribution in the vasculature. We have already demonstrated the expression of LOXL2 in endothelial cells of growing capillaries during development and postischemic revascularization of the hind limb (9). We here detected LOXL2 in vessels of the tibialis anterior muscle, both in capillaries and arterioles where it displayed a comparatively strong staining in the media (**Fig. 8*A***). LOXL2 was also detected in the aorta, both in smooth muscle cells of the media and in endothelial cells of the intima, with a similar pattern as LOX (Fig. 8*B*). Furthermore, both proteins were detected in aorta lysates by Western blot analysis (Fig. 8*C*). Sequential extraction of proteins by Triton X-100 followed by SDS solubilization and reduction of the insoluble material allowed detection of LOX and LOXL2 in the same fraction as fibronectin (Fig. 8*C*).

## DISCUSSION

We here provide nanoscale structural data describing the organization of a mammalian member of the LOX family using recombinant human FL-LOXL2 purified from the secretion medium of eukaryotic cells. We show that LOXL2 is organized as a stalk composed of SRCR14, exposing the catalytic domain at its tip. We observed similar structures using the independent approaches of SAXS and EM. Our data suggest that SRCR domains 1 and 2 are located away from the catalytic domain, supporting the notion that LOXL2 processing is not associated with enzyme activation, as recently proposed in López-Jiménez *et al.* (4). Such a structural organization nevertheless fits the recently described processing of LOXL2 between SRCR domains 2 and 3. Indeed, the string-of-pearls organization of the SRCR domains and the flexibility of the 23-residue linker between SRCR2 and SRCR3 supports the exposure of a cleavage site in this linker and thus the release of the N-terminal domains 1 and 2 upon cleavage. Cleavage of LOX and LOXL1 participates in enzyme activation through processing by BMP-1 (29–31). We show here that this protease is not able to process LOXL2. On the other hand, LOXL2 processing at Arg-Phe-Arg-Lys (RFKR) by a serine protease was recently described in López-Jiménez *et al.* (4), in agreement with the inhibition of LOXL2 processing by serine protease inhibitors as reported here. Our data also fit with the crystal structure of a truncated and inactive LOXL2 enzyme that was recently reported in Zhang *et al*. (24). In addition, comparing both structures suggests that a conformational change could occur upon cleavage of the 2 N-terminal domains, bringing SRCR domain 3 closer to the catalytic site. Such a change would not be associated with enzyme activation but could modulate substrate binding, as suggested for collagen IV (4).

The association of LOXL2 with *ELN* as a susceptibility gene for glaucoma was already proposed in Urban *et al.* (34), but no functional interaction between these proteins was demonstrated in this study. More recently, the identification of LOXL2 mutations in patients suffering from middermal elastolysis directly raised the possibility that LOXL2 could participate in elastin cross-linking (14). We show here the direct binding of LOXL2 to TE using surface plasmon resonance, and we could detect lysine oxidation by recombinant LOXL2. Oxidation of soluble TE by LOXL2 had already been suggested in Xu *et al.* (35). Furthermore, deleting SRCR domains 1–3 had no impact on such activity, which further supports that FL-LOXL2 does not require activation and is fully active on this substrate. There was, however, no experimental data demonstrating the cross-linking of TE by LOXL2 [reviewed by Moon *et al.* (2)]. We show here for the first time that TE is cross-linked into an insoluble, gel-like material under the influence of LOXL2 *in vitro*. The much lower rate of lysine modification in cTE compared with mature elastin generated *in vivo* could result from either the *in vitro* experimental conditions or the lack of elastin-associated proteins such as fibulins. Alternatively, the limited cross-linking of TE by LOXL2 detected at 37°C could indicate that this enzyme could only be a minor player in the armament of elastin cross-linking enzymes. Physicochemical characterization of cTE in comparison with isolated aortic elastin revealed that cTE shares similarities but also differences with mature elastin. The cross-links dehydrolysinonorleucine (ΔLNL), desmosine, and isodesmosine were found in cTE; however, they were at lower abundance compared with mature elastin, indicating that cTE is less or differently cross-linked. cTE nevertheless clearly exhibits elastic properties and a reduced elastic modulus of the same order of magnitude as for elastin. However, the slightly higher stiffness and lower *T*_g_ of cTE indicate differences in the organization of the 2 materials. With respect to susceptibility toward enzymatic cleavage, cTE exhibits significant resistance to trypsin digestion, whereas non-cTE is readily hydrolyzed. Because there are very few arginine residues in mature elastin and most of the lysine residues are involved in covalent cross-linking in mature elastin (36), it is virtually completely resistant to trypsin. cTE was, however, partly cleaved by trypsin at longer incubation times, suggesting that not all lysine residues present in cTE are fully involved in cross-linking. It is worth mentioning that the cTE generated by LOXL2 exhibited a much higher resistance against trypsin in comparison with the elastin-like biomaterial formed after exposure of TE to an amine oxidase from *Aspergillus nidulans*, which was fully degraded by trypsin after 8 h in similar experimental conditions (28). Modulating the cross-linking conditions such as temperature, TE:LOXL2 ratio, and incubation time could further enable tuning of the cTE characteristics, including proteolytic resistance and mechanical properties.

Whereas inactivation of both *LOX* and *LOXL1* genes affects elastin cross-linking, these effects are only partial, as measured by the amount of desmosine in the lungs of LOX^−/−^ mice (6) and of LOXL1^−/−^ mice (7). These data suggest that elastin cross-linking does not result from the activity of a single member of the LOX family. Elastin synthesis is a multistep process comprising coacervation of TE, microfibrillar deposition, and cross-linking. The order of these events is not completely understood, and it has been proposed that cross-linking could precede deposition (37). Whereas coacervation of TE has been extensively studied *in vitro* over the past 30 yr (38), investigation of cross-linking has been limited because of the lack or limited availability of LOX enzymes. *In vitro* studies have demonstrated that self-assembly results from interactions between the hydrophobic domains of TE and that polymerization results from cross-linking of lysines present in the hydrophilic domains. Cross-linking provides both structural restrictions and stability to elastic fibers, together with insolubility and protease resistance (39). Stabilization of intermediate states through cross-linking could also participate in tuning elastin organization. Indeed, the importance of lability of the hydrophilic cross-linking domains for coacervation was recently demonstrated in Reichheld *et al.* (40), possibly by preventing inappropriate aggregation. Such a function could be achieved by the intradomain cross-links generated by LOXL2 that we have detected. Indeed, analysis of cross-links in native elastin demonstrated the presence of many intramolecular cross-links resulting in an unordered network of TE molecules (41). This hypothesis thus raises the possibility that LOXL2 participates in some steps of elastogenesis together with other LOXs. The recent identification of patients suffering from middermal elastolysis also supports the direct involvement of LOXL2 in the stability and maintenance of elastin (14).

LOX inactivation results in perinatal aorta rupture, with 61% inhibition of elastin cross-links in the aorta, whereas LOXL1 has no effect on aorta development, suggesting regulation of elastogenesis and cross-linking in a tissue-specific manner. An interesting hypothesis could be that LOXL2 participates in elastin cross-linking in the vascular wall. Inactivation of the LOXL2 gene is lethal in 50% of the newborn mice as a result of congenital heart defects and results in vascular defects in the surviving animals, but no substrate has been identified to be involved in this phenotype up to now (42). We could detect the localization of LOXL2 in the vascular wall of arterioles and the aorta, with a similar distribution as already described for LOX in Liu *et al.* (7). Expression of LOXL2 in the wall of small arteries had already been described in Csiszar (43). More recently, LOXL2 was detected in retina arterioles, with increased expression in growing microaneurysms (44).

Finally, in addition to raising the possibility that LOXL2 is involved in elastin cross-linking *in vivo*, our study allowed characterization of LOXL2 as a biomimetic tool that should be considered for the generation of elastin as a biomaterial. Indeed, whereas chemical cross-linking is often associated with toxicity that limits its use for cell-based therapies in tissue engineering, a biomimetic approach based on LOXL2-mediated cross-linking could improve the mechanical properties of elastin-based biomaterials without affecting cell survival.

## Supplementary Material

This article includes supplemental data. Please visit *http://www.fasebj.org* to obtain this information.

## Abbreviations

β-APN: β-aminopropionitrile
3D: 3-dimensional
AFM: atomic force microscopy
AUC: analytical ultracentrifugation
BMP-1: bone morphogenetic protein-1
CD: circular dichroism
CG: cathepsin G
CHO: Chinese hamster ovary
cTE: cross-linked TE
DHFR: dihydrofolate reductase
ECM: extracellular matrix
EM: electron microscopy
FL-LOXL2: full-length LOXL2
LC: liquid chromatography
LOX: lysyl oxidase
LOXL: LOX-like
MS: mass spectrometry
MS/MS: tandem MS
PCPE-1: procollagen C-proteinase enhancer 1
PE: pancreatic elastase
*R*_g_: radius of gyration
SAXS: small-angle X-ray scattering
SRCR: scavenger receptor cysteine-rich
SRCR14: the 4 SRCR domains
TE: tropoelastin
*T*_g_: glass transition temperature
