# Supplementary material for: Lysyl oxidase–like 2 (LOXL2)–mediated cross-linking of tropoelastin
Source: FASEB J. 2019 Jan 24;33(4):5468–81. doi: 10.1096/fj.201801860RR (PMC6629125; doi:10.1096/fj.201801860RR)
Supplement: Supplementary file 1 [file fj.201801860RR.sd1.docx]

**SUPPLEMENTAL FIGURE LEGENDS**

**Fig. S1: Proteolytic processing of LOXL2.**

**A** CHO cells overexpressing human recombinant LOXL2 were cultured in the presence of protease inhibitors (AEBSF, or aprotinine or leupeptine) at the indicated concentrations (µM). Talon resin was used for rapid batch-concentration of LOXL2 from 100 µL of the secretion medium. Proteins were analysed by SDS-PAGE under reducing conditions followed by Coomassie Blue staining.

**B** CPIII-long (positive control, 400 nM), or purified human recombinant LOXL2 (300 nM) were incubated in the presence or absence of BMP-1 (40 nM) alone or with PCPE-1 (400 nM) for 2h at 37 °C in assay buffer consisting of 50 mM Hepes pH 7.4, 150mM NaCl, 5 mM CaCl_2_. Proteins were analyzed by SDS PAGE under reducing conditions followed by Coomassie Blue staining.

**Fig. S2: Characterization of recombinant LOXL2 and SRCR14.**

**A** LogI versus q with Gnom fit.

**B** Distance distribution function P(R).

**C** Guinier plots with residuals.

**D** Best fit rigid body model of SRCR14 from Coral compared to the experimental data.

**E** Normalised Kratky plot for LOXL2 and SRCR domains shows that both proteins are elongated and non-globular as the peak for globular proteins would fall on the cross-hairs (the globularity point).

**F** Flexibility plots q^4^ (Porod-Debye) and q^3^ (SIBYLS). Both proteins plateau on the q^3^ plot but not the q^4^ plot which indicate they are both flexible.

**Fig. S3: LOXL2-mediated modification of tropoelastin lysines.** The chart shows the extent of modification of Lys residues measured by amino acid analysis. While all Lys residues in tropoelastin are unmodified, 90% of all Lys residues are modified in mature elastin. The incubation of tropoelastin with LOXL2 resulted in up to 14% of modified Lys depending on experimental conditions. Experiments were carried in duplicates.

**Fig. S4: Impact of LOXL2-mediated cross-linking on the physical properties of tropoelastin**.

**A** DSC thermograms of cTE (red solid line: 2nd heating run, red dashed line: 3rd heating run) and bovine elastin (black solid line: 2nd heating run, black dashed line: 3rd heating run). The T_g_ was found to be around 160 °C and around 180 °C for cTE and bovine elastin, respectively.

**B** Swelling behavior of cTE in PBS (red filled square) or doubly distilled water (red empty square) and bovine elastin in PBS (black filled circle) and doubly distilled water (black empty circle). cTE takes up 6 – 8 times more water as compared to bovine elastin.
