# Supplementary material for: Lysyl oxidase–like 2 (LOXL2)–mediated cross-linking of tropoelastin
Source: FASEB J. 2019 Jan 24;33(4):5468–81. doi: 10.1096/fj.201801860RR (PMC6629125; doi:10.1096/fj.201801860RR)
Supplement: Supplementary file 2 [file fj.201801860RR.sf1.pdf]

FIG S1

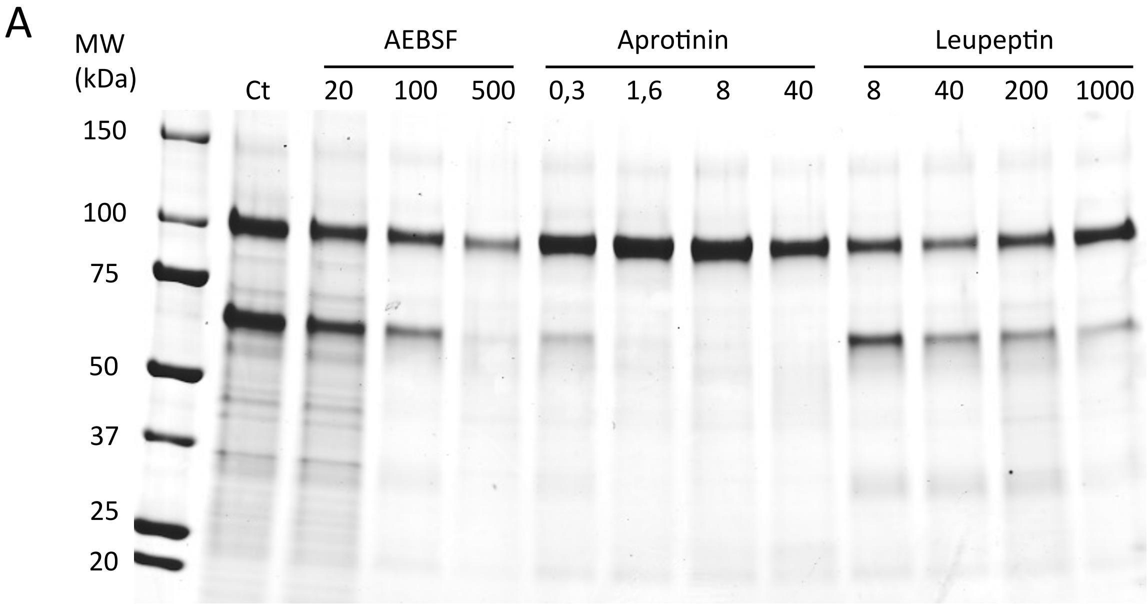
A
MW (kDa)
Ct
AEBSF
20
100
500
Aprotinin
0,3
1,6
8
40
Leupeptin
8
40
200
1000
150
100
75
50
37
25
20

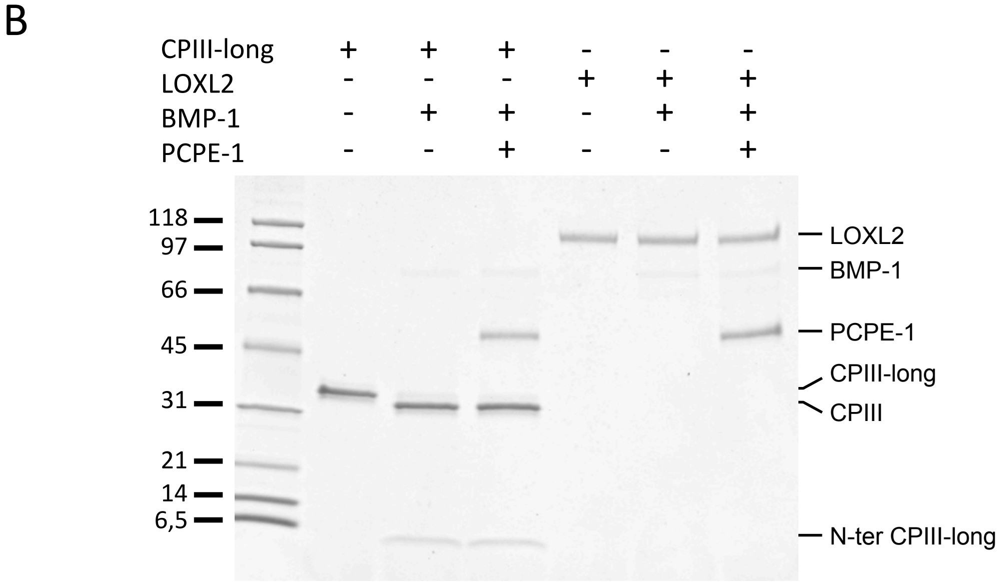
B
CPIII-long + + + - - -
LOXL2 - - - + + +
BMP-1 - + + - + +
PCPE-1 - - + - - +
118
97
66
45
31
21
14
6,5
LOXL2
BMP-1
PCPE-1
CPIII-long
CPIII
N-ter CPIII-long

FIG S2

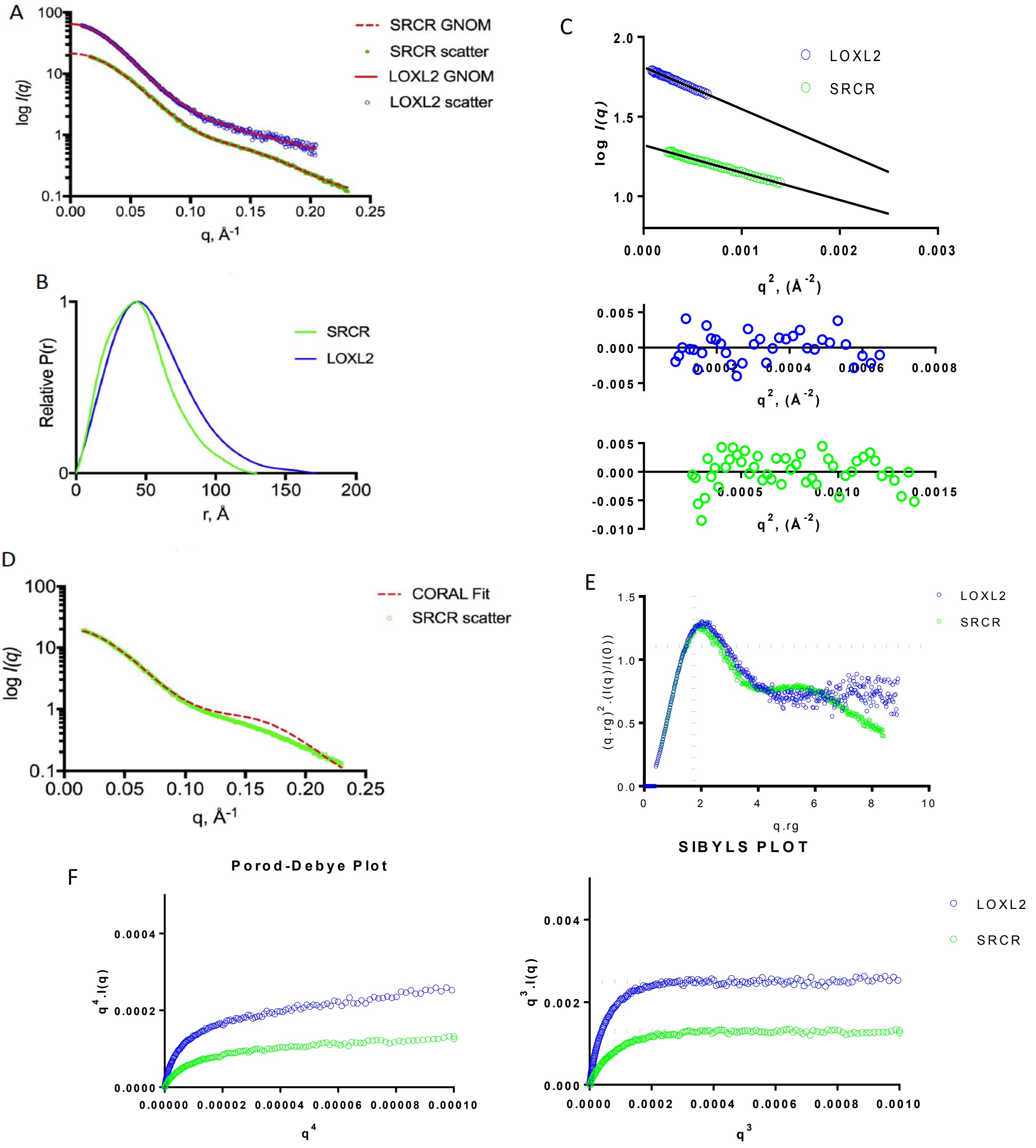
A
SRCR GNOM
SRCR scatter
LOXL2 GNOM
LOXL2 scatter
log I(q)
q, Å-1
B
SRCR
LOXL2
Relative P(r)
r, Å
C
LOXL2
SRCR
log I(q)
q2, (Å-2)
D
CORAL Fit
SRCR scatter
log I(q)
q, Å-1
E
LOXL2
SRCR
(q.rg)2.(I(q)/I(0))
q.rg
SIBYLS PLOT
F
Porod-Debye Plot
q4.I(q)
q4
LOXL2
SRCR
q3.I(q)
q3

FIG S3

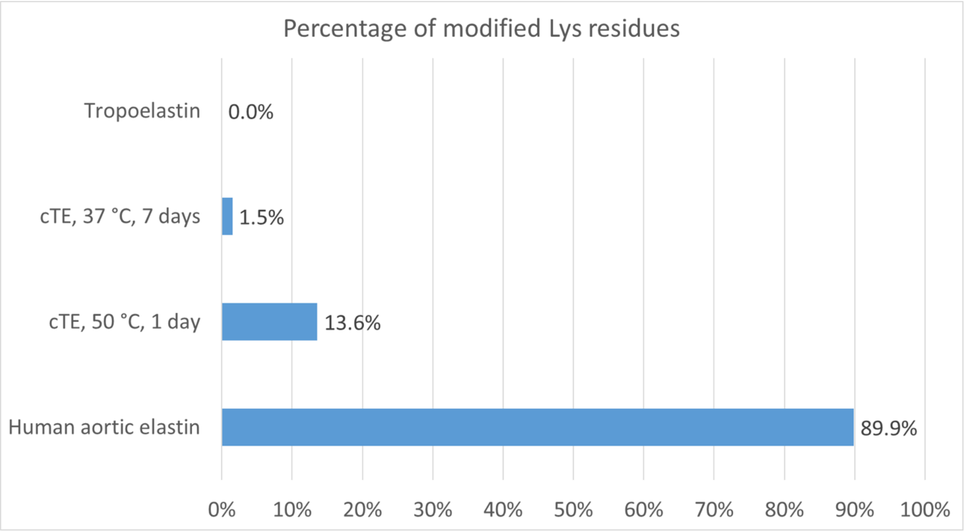
Percentage of modified Lys residues
Tropoelastin
0.0%
cTE, 37 °C, 7 days
1.5%
cTE, 50 °C, 1 day
13.6%
Human aortic elastin
89.9%
0%
10%
20%
30%
40%
50%
60%
70%
80%
90%
100%

FIG S4

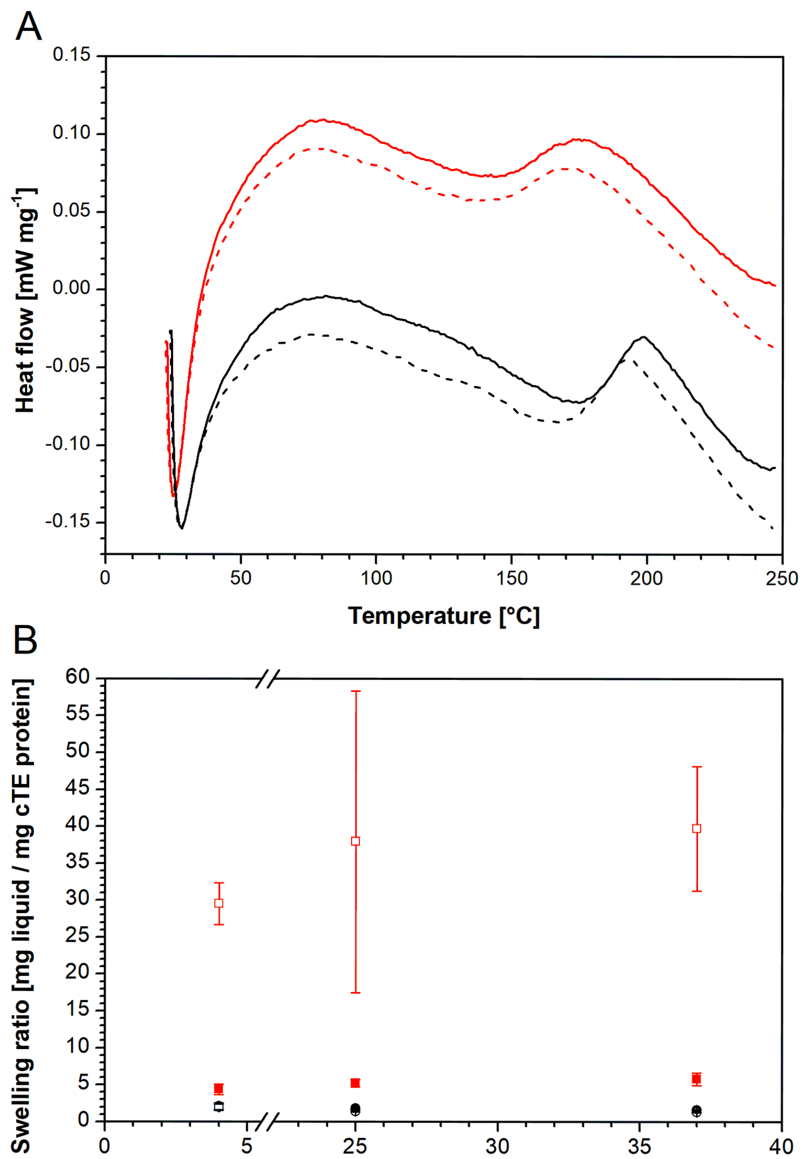

A
0.15
0.10
0.05
0.00
-0.05
-0.10
-0.15
Heat flow [mW mg^{-1}]
0
50
100
150
200
250
Temperature [°C]
B
60
55
50
45
40
35
30
25
20
15
10
5
0
Swelling ratio [mg liquid / mg cTE protein]
0
5
25
30
35
40
Temperature [°C]

SUPPLEMENTARY MOVIE

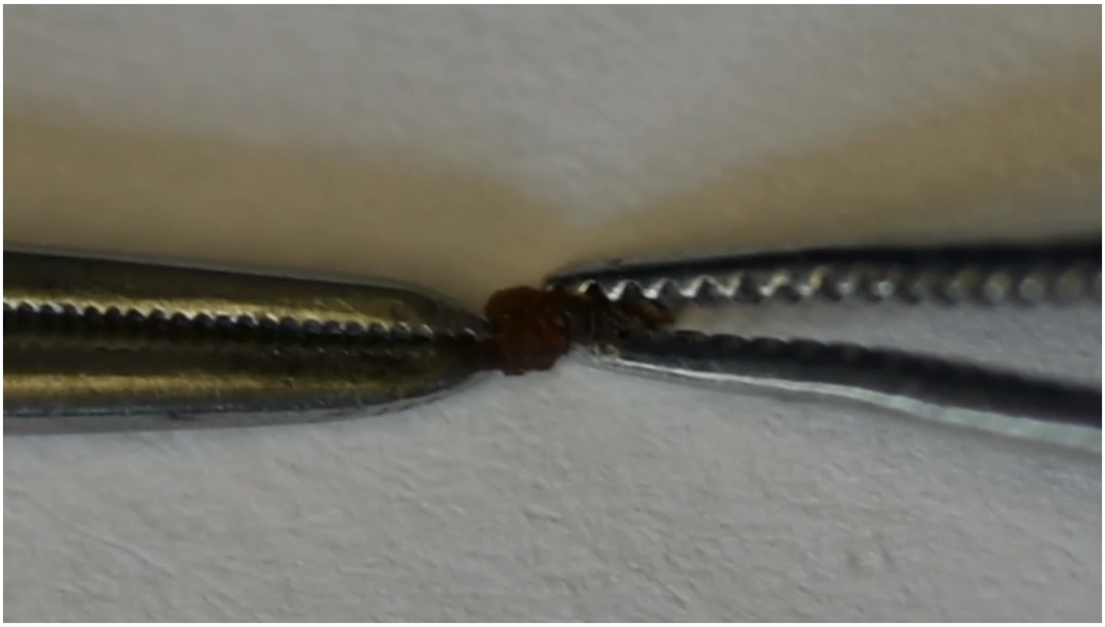

Movie 1 : The stretching process of a pellet of LOXL2-induced in vitro-cross-linked human tropoelastin demonstrates the elasticity of the material.
